# Quality Program: what influences the opinion of nursing team

**DOI:** 10.1590/S1679-45082016GS3615

**Published:** 2016

**Authors:** Fernanda Mazzoni da Costa, Irene Duarte Souza, Maria Inês Monteiro, Maria Helena Baena de Moraes Lopes, Rosangela Maria Greco

**Affiliations:** 1 Hospital Universitário, Universidade Federal de Juiz de Fora, Juiz de Fora, MG, Brazil.; 2 Universidade Estadual de Campinas, Campinas, SP, Brazil.; 3 Universidade Federal de Juiz de Fora, Juiz de Fora, MG, Brazil.

**Keywords:** Health Management, Quality Management, Working conditions, Nursing staff, hospital, Quality of health care, Nursing, team

## Abstract

**Objective:**

To analyze the major impact variables in the opinion of nursing staff about the Quality Program of a teaching hospital.

**Methods:**

An exploratory-descriptive study was performed with 72 nursing staff. Data were collected through self-administered questionnaire containing 24 statements about the Quality Program; and the degree of agreement of the participants was expressed in a Likert scale. The collected data were analyzed by factor analysis and Pearson’s correlation coefficient.

**Results:**

The analysis grouped the statements in six factors. The Pearson’s correlation coefficient defined a scale of influence of the variables within each factor, whose variable with the greatest impact in each factor is the priority issue for improving worker opinion about the Quality Program. The priority variables were to believe the Quality Program contributes to the hospital; to understand the program orientations; interest in hospital quality direction; and do not feel exhausted due to the program.

**Conclusion:**

These variables must be focused during the implementation and execution of Quality Program, as they have greater impact on improving opinion regarding the Quality Program and thus helping to increase compliance of the nursing staff to the program.

## RESUMO

**Objetivo:**

Analisar as variáveis de maior impacto na opinião dos trabalhadores de enfermagem sobre um Programa de Qualidade de um hospital de ensino.

**Métodos:**

Estudo exploratório-descritivo, desenvolvido com 72 trabalhadores de enfermagem, com dados coletados por meio de questionário autoaplicável, contendo 24 afirmações com escala Likert sobre o Programa de Qualidade. Para análise dos dados, foram utilizados análise fatorial e coeficiente de correlação de Pearson.

**Resultados:**

A análise agrupou as afirmações em seis fatores. O coeficiente de correlação de Pearson definiu uma escala de influência das variáveis dentro de cada fator, cuja variável de maior impacto em cada fator representa a questão prioritária para a melhoria da opinião do trabalhador sobre o Programa de Qualidade. As variáveis prioritárias foram: acreditar que o Programa de Qualidade contribui para o hospital; compreender as orientações do programa; interessar-se pelos rumos de qualidade do hospital; e não se sentir desgastado em função do programa.

**Conclusão:**

Estas variáveis devem ser focadas durante a implantação e a execução de um Programa de Qualidade, pois possuem maior impacto na melhoria da opinião em relação a ele, contribuindo para aumentar a adesão dos trabalhadores de enfermagem.

## INTRODUCTION

Healthcare organizations have gone through rapid and deep transformation, aiming to meet the demands of an ever-challenging clientele.^(1)^ From this perspective, some healthcare service managers have used some practices with high standard of quality and safety to gain social recognition.^(2)^ Quality management in health is opposed to unsafe care, which may result in avoidable morbidity and mortality, in addition to expenses with maintenance of patients in health systems.^(3)^ The scope of standards, understood as a basis for enhancing quality, is the main conductor of safety efforts. Internationally, quality is a concern, especially regarding patient safety.^(4)^

The patient safety goals defined by the World Health Organization (WHO), in 2004, stand out in the recent international history.^(5)^ In Brazil, the theme has gained importance as from 2012, with the establishment of the Quality and Patient Safety Technical Chamber by the Ministry of Health, which is mandatory at all federal hospitals.^(6)^In the State of Minas Gerais (MG), *e.g*., we point out the Program for Quality Strengthening and Improvement at the hospitals from the National Public Health System.^(7)^

Within this context, the present study had the following guiding question: which of the significant variables most influence the opinion of nursing professionals as to the Quality Program, favoring their compliance?

## OBJECTIVE

To analyze the variables of greatest impact on the opinion of nursing professionals about a Quality Program in a teaching hospital.

## METHODS

This is an exploratory-descriptive study developed by means of a survey of the opinions of nursing professionals about the Quality Program of a medium-sized teaching hospital, accredited for providing medium- and high-complexity care, in a large city in the interior of Minas Gerais (MG).

The hospital where this study was carried out had a Quality Management Center, established in 2009, and the Total Quality Program was under development.^(8)^ After three years of the program, low compliance of the employees was noted. Although most workers believed the program had a positive influence on their conditions and interpersonal work relations, and had agree with it, an expressive percentage of professionals was unaware of the implications of a Quality Program, and did not feel included in what had been developed at the organization.^(9)^

Out of 278 nursing professionals from the organization, 82 were selected for a random sampling, based on the variance of the responses of the pilot sample of 12 individuals, taking into consideration a maximum admissible error of 5%, a 95% confidence interval, and a minimal addition of 15% as a safety margin for replacing sample losses. The study included workers that were involved in direct care or in administrative services linked to nursing, and excluded those that did not wish to participate and/or did not sign the Informed Consent Form.

Data was collected between May and July 2012, with a self-applied questionnaire developed for the study, based on literature and on the investigators’ experience. It was pretested on ten subjects of the target population, who evaluated the questionnaire regarding its content, format, and structure. The adjustments resulted in an instrument comprising 11 questions for sociodemographic characterization of participants, and 24 statements on the Quality Program of the organization. The degree of agreement of the participant should be expressed on a scale of response amplitude, which included five levels, as follows: “totally agree”, “partially agree”, “I have no opinion on that”, “partially disagree”, and “totally disagree”. The Cronbach α coefficient (0.878) demonstrated adequate internal consistency of the instrument.

Ten questionnaires were excluded for being incomplete. The data from the remaining 72 questionnaires were processed using the Statistical Package Social Sciences (SPSS) program, version 14.0, and submitted to exploratory factorial analysis. To obtain the position of each variable in each factor, a correlation matrix was prepared, with extraction of the initial factors, octagonal rotation by the Varimax method, and calculation of the factorial scores. The Bartlett sphericity test (1004.348) and the Kaiser-Meyer-Olkin index (0.820) indicated that the sample was adequate for factorial analysis, which grouped the statements into six factors (work conditions: evaluates to what extent the employees believes that the Quality Program interferes in their work conditions; agreement: assesses agreement/approval of the Quality Program; belonging: quantifies the feeling of belonging of the workers regarding the program; tranquility: measures how much easeness the employees believe they have to develop their activities; interpersonal relations: verifies the perception of the professionals as to the interference of quality in relationships; and personal life: investigates the workers’ perception about the interference of quality in their life).

The strength of association among the variables of each factor was analyzed by means of Pearson’s correlation coefficient. Hence, the influence of each variable on the individual’s opinion relative to the factor to which it was linked was noted. According to the coefficient, all variables are correlated in the same direction (positive) as to the factorial analysis grouping of the similar variables in the same factor. Thus, the variables with a coefficient near one represent a strong correlation with the factor and a high impact of the variable as to the factor, that is, they have a greater potential of influencing the individual’s opinion, so that he/she evaluates positively the factor under analysis. Whereas the variables with a coefficient near zero show a weak correlation and consequently, interfere very little in the individual’s opinion about that factor. Based on the findings of this test, a scale of influence of the variables on each fact was prepared. The variable with greatest impact on the factor represents the priority question in actions that aim at improving the workers’ opinion about the factor to which it is correlated, and therefore, to the program as a whole.

The study was approved by the Research Ethics Committee of the *Universidade Federal de Juiz de Fora*, Juiz de Fora (MG), Brazil, under number 262/2010, CAAE: 0171.0.180.00-10.

## RESULTS

Among the study participants, 78% were women; 63% were aged between 30 and 49 years; 53.5% had graduated more than six years before; 54.6% had been working for at least six years at the organization; 31.7% had more than one job; and 54.9% were hired according to the Consolidated Labor Laws.

Chart 1 shows the scale of influence of the variables in each factor, demonstrating the priority issues for improving the opinion of workers about the factors analyzed, developed with a basis on the findings of Pearson’s correlation coefficient.

**Chart 1:** Scale of influence of the variables on each factor

Factors	Pearson’s correlation coefficient	Variables by order of influence on the factor
Factor 1: work conditions	0.837	Belief that the Quality Program contributes towards the hospital’s provision of necessary orientation and resources for carrying out the work
	0.717	Belief that the hospital is concerned with their health and well being
	0.663	Belief that the Quality Program promotes greater quality of life at work
	0.587	Belief that the Quality Program makes the work motivating
	0.579	Belief that the effort to meet the Quality Program demands is recognized by the hospital
	0.571	Belief that the Quality Program contributes towards safety in carrying out tasks
	0.524	Belief that the Quality Program facilitates work
	0.483	Perception that the Quality Program brings care closer
Factor 2: agreement	0.732	Understanding the orientations of the Quality Program
	0.716	Possibility of carrying out the orientations regarding quality
	0.677	The hospital adjusts requirements in the Quality Program relative to benefits it offers
	0.605	Agreement with the conduction of the Quality Program
	0.550	Belief that the hospital offers the resources necessary to meet the demands of the Quality Program
	0.508	Knowledge of the history of quality in the organization
Factor 3: belonging	0.799	Interest in the trends in quality at the hospital
	0.797	Feeling that he/she is really a part of the hospital’s conquests
	0.780	Feeling of responsibility for quality at the hospital
	0.628	Perception of being involved with the Quality Program
Factor 4: tranquility at work	0.773	Not feeling worn out because of the Quality Program
	0.758	Considering that he/she could advise a friend to come work at the hospital
	0.538	Feeling natural in following the orientations of the Quality Program
	0.512	Creativity is stimulated

In performing the factorial analysis, only one variable was attributed to factor 5 (interpersonal relations at work) and one to 6 (personal life). Since these factors are attributed to only one variable, it is not possible to construct a scale of influence of the variables within these factors. The correlation of a single variable with itself would result in erroneous maximal correlation strength; therefore, these two factors were not submitted to Pearson’s correlation test, but were analyzed based on the variables attributed to them.

## DISCUSSION

Knowledge about the order of influence of the variables on the opinion of employees provides subsidies that guide the manager when making strategic and operational decisions to implement actions on the variable representing the priority issue for improving employee opinion about each factor, and consequently, about the Quality Program, contributing to increase their compliance with the program.

The priority issue for the employees in factor 1, work conditions, is “Belief that the Quality Program contributes towards the hospital’s provision of necessary orientation and resources for executing the job”.

It is interesting to note that workers consider having the necessary orientations and resources for the adequate development of their jobs brings collective benefits, such as cooperation with the multidisciplinary team, promotion of qualified care, and consonance with the objectives of the organization. The priority for them was to give a good evaluation of the factor and the program, to the detriment of other issues that would bring individual benefits, such as those related to their health, safety, motivation, recognition, and ease in carrying out tasks, and quality of life on the job.

Some studies showed in organizations that have Quality Programs, the employees feel better prepared for performing their activities, since they have standardized routines, greater work organization, in addition to the material, technical, and human resources needed.^(2)^ Moreover, in the opinion of nurses, implementing Quality Programs promotes organization of the service and optimization of processes, favoring safe work by the team and providing time for systematization of nursing care.^(8)^ All these facts demonstrate the results of care are related to the conditions to carry out the activities, and to the concern about having the appropriate resources to deliver qualified care.

If on the one hand these results show that workers understand that the necessary orientations and resources for performing their tasks are determinant for their performance, on the other hand, they may mean that the workers do not consider themselves beneficiaries of Quality Programs. Studies demonstrated the professionals more clearly perceive the advantages of Quality Programs for patients than for the other stakeholders, including the workers.^(10)^

The satisfaction of the team with working conditions directly affects the quality of care given and patient satisfaction.^(11)^ Studies identified that in better working environments, the level of personnel and of qualification were associated with more positive work experiences, and with less concern about quality of care by the professionals, with a significantly lower risk of death and disability of patients.^(12)^ The authors further highlighted the positive effects of the increased number of nurses on nursing care results were directly proportional to quality of the working environment.^(13)^ Thus, despite the professional’s qualifications, their performance is circumscribed by working conditions, which demonstrates the importance of the organization making an effort to provide the necessary resources for appropriate conduction of the job, so that better results are achieved.

In factor 2, agreement, a primary issue for the employees was “Comprehension of the Quality Program orientations”.

The success in implementing Quality Programs depends on individual and collective efforts.^(14)^ However, although many professionals consider them as a tool capable of intervening in the work process and providing safety, few effectively understand the process and their responsibility.^(8)^ Many professionals mechanically assimilate the information about Quality Programs, without developing a reflexive and critical practice.^(10)^

Understanding of the program orientations is important for the workers to feel motivated to comply with the actions proposed, since people do not get involved with what they do not understand.^(15)^ Implementing Quality Programs, not incorporating the fundamental concepts by means of complete, clear, objective and uniform information, hinders comprehension of the process and is a great barrier in employee engagement.^(16)^

The organizations should strive to overcome this obstacle by means of adequate communication. Considering the importance of training employees, some participatory learning strategies in permanent health education have been developed, aiming to include the subject in the process through collective reflection on the practice, appreciating the worker by recognizing their knowledge and promoting team work with decision-making by consensus.^(17)^

As a priority issue in factor 3, belonging, the workers elected *“*Interest in the quality trends of the hospital”. Although the employees recognize the importance of participating in the Quality Programs, many admit that they do not get involved with the proposal.^(8)^

Quality of life at work and the standard of work carried out are influenced by issues associated with family and social relations, and with the worker’s routine, including the feeling of belonging.^(18)^ To feel as part of the process is essential for subjects to engage in movements, in which practices and meanings are updated. This notion of belonging depends on the existence of shared management areas that allow collective construction of values and meaning for production of health.^(19)^

The nursing team accredited by the Magnet Recognition Program^®^ from the United States reports more opportunities to have its opinion received on issues related to the work space and participation in shared leadership, as well as an environment with a positive atmosphere.^(20)^

There are studies showing that employees feel proud, satisfied, and acknowledged when they perceive they were important in obtaining certification resulting from implementation of Quality Programs and in working at accredited services.^(1)^ Since humans are gregarious and needs to be recognized by the other, by affirming their belonging to a social group,^(21)^ this feeling of pride comes from the notion of belonging to an organization for which one has admiration and value identification.^(22)^

As priority issue in factor 4, tranquility at work, employees point out that *“*they do not feel worn out because of the Quality Program”. Consequently, if the worker feels exhausted because of the Quality Program or of the level of institutional requirements, he/she has a negative opinion as to tranquility on the job in reference to the program as a whole.

The exhaustion of the employee is a result of stressful situation in day-to-day work. Most research frequently links stress to the work site/environment and the characteristics of the professional job, such as emergency, operating room, and intensive therapy unit.^(23,24)^

A comparative study based on the perception of the nursing team members of hospitals – accredited or not accredited - analyzes how the existence of a Quality Program can wear out or protect the team. Work at an accredited hospital was a protective factor against stress when considering the following situations: stress due to lack of materials, lack of human resources, and work in inadequate physical environments. However, working at an accredited hospital was a risk factor for stress because of the fact of having to reconcile professional and family issues, care for severely ill patients, help family members, and manage or supervise the work of others. In summary, working at an accredited hospital protects from the stress related to material, human, and physical resources, but it is an adverse factor for emotional health of nurses when relational aspects and the complexity of the job are considered.^(25)^

In order to motivate tranquility of workers and consequently, compliance with the Quality Program, the program itself cannot be perceived as a stressing factor that leads to exhaustion.

Based on a factorial analysis, factor 5 had just one question: “Does the Quality Program promote a better interaction with my work colleagues and managers?” Interpersonal relationships at work can be a stressful factor, more frequent in accredited hospitals than in those that are not accredited. For nurses, interpersonal relations are especially stressful in situations in which they have to reconcile professional and family issues; assist severely ill patients, and especially, help family members.^(25)^ Therefore, the Quality Program should adopt strategies to promote interaction between the multidisciplinary team, patients and family members, as well as to minimize hierarchy-/vertical-based structures and communication barriers.

Factor 6 - personal life – had only one variable: “During moments of leisure and rest, I am capable of forgetting the demands of the Quality Program”. Frequently, work conditions generate organic and psychological problems resulting from stress and exhaustion, with repercussions in personal life.^(26)^ This result corroborates the reflection as to factor 4, whose program could not be seen as a stressful factor that compromises one’s personal life or even leisure and rest of the nursing employees.

## CONCLUSION

The variables that impacted most on the opinion of nursing employees about the Quality Program studied were: belief that the Quality Program contributes towards the hospital’s provision of necessary orientations and resources for execution of the job; comprehension of the orientation of the Quality Program; interest in the trends of quality of the hospital; condition of not feeling worn out because of the Quality Program; promoting better interaction with work colleagues and managers; and capacity to forget the demands of the Quality Program during moments of leisure and rest.

This information enables preparing strategies to improve the program based on the views of the employees, favoring their compliance. Therefore, the study provides the priority variables for strategic decision-making by the manager, in order to improve the opinion of the nursing team about the Quality Program and, consequently, afford greater compliance with the program, seeking greater qualification in care. Additionally, it enhances the discussions and reflections about participation of nurses in the Quality Program.
